# Diacetatobis(propane-1,3-diamine)nickel(II) dihydrate

**DOI:** 10.1107/S1600536810010172

**Published:** 2010-03-24

**Authors:** Islam Ullah Khan, Ejaz, Onur Şahin, Orhan Büyükgüngör

**Affiliations:** a Materials Chemistry Laboratry, Department of Chemistry, GC University, Lahore 54000, Pakistan; b Department of Physics, Ondokuz Mayıs University, TR-55139 Samsun, Turkey

## Abstract

In the title complex, [Ni(CH_3_COO)_2_(C_3_H_10_N_2_)_2_]·2H_2_O, the Ni^II^ atom resides on a centre of symmetry and is in an octa­hedral coordination environment comprising four amino N atoms and two carboxyl­ate O atoms. Inter­molecular N—H⋯O and O—H⋯O hydrogen bonds produce *R*_2_^1^(6), *R*_2_^2^(12), *R*_3_^2^(8) and *R*_5_^5^(16) rings, which generate a two-dimensional polymeric network parallel to (001).

## Related literature

For the graph-set analysis of hydrogen-bond patterns, see: Bernstein *et al.* (1995 ▶). For details of ring puckering analysis, see: Cremer & Pople (1975 ▶). For the effect of hydrogen bonding on the coordination in *trans*-di(salicylato)bis­(1,3- diamino­propane-*N,N*’)copper(II), see: Sundberg *et al.* (2001 ▶).
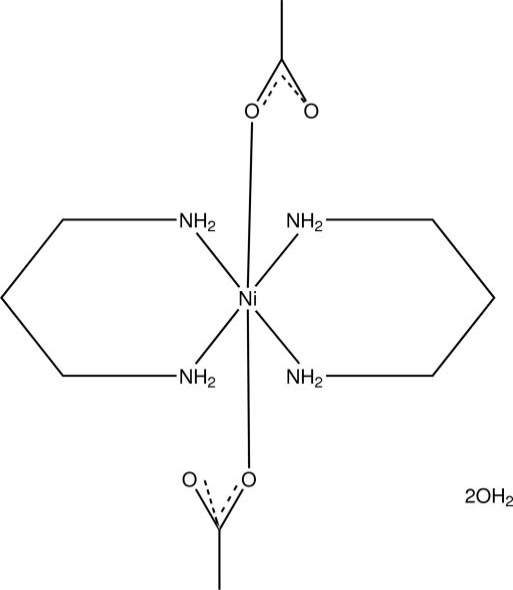


## Experimental

### 

#### Crystal data

[Ni(C_2_H_3_O_2_)_2_(C_3_H_10_N_2_)_2_]·2H_2_O
*M* *_r_* = 361.09
Triclinic, 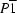

*a* = 6.6268 (3) Å
*b* = 7.8164 (3) Å
*c* = 8.9123 (4) Å
α = 73.933 (2)°
β = 80.797 (3)°
γ = 75.323 (2)°
*V* = 427.12 (3) Å^3^
*Z* = 1
Mo *K*α radiation
μ = 1.17 mm^−1^
*T* = 296 K
0.32 × 0.18 × 0.13 mm

#### Data collection

Bruker Kappa APEXII CCD area-detector diffractometer
Absorption correction: multi-scan (*SADABS*; Bruker, 2009 ▶) *T*_min_ = 0.775, *T*_max_ = 0.857
7230 measured reflections
2078 independent reflections
2021 reflections with *I* > 2σ(*I*)
*R*_int_ = 0.025

#### Refinement

*R*[*F* ^2^ > 2σ(*F* ^2^)] = 0.024
*wR*(*F* ^2^) = 0.094
*S* = 1.02
2078 reflections
122 parameters
3 restraints
H atoms treated by a mixture of independent and constrained refinement
Δρ_max_ = 0.53 e Å^−3^
Δρ_min_ = −0.58 e Å^−3^

### 

Data collection: *APEX2* (Bruker, 2009 ▶); cell refinement: *SAINT* (Bruker, 2009 ▶); data reduction: *SAINT*; program(s) used to solve structure: *SHELXS97* (Sheldrick, 2008 ▶); program(s) used to refine structure: *SHELXL97* (Sheldrick, 2008 ▶); molecular graphics: *ORTEP-3 for Windows* (Farrugia, 1997 ▶); software used to prepare material for publication: *WinGX* (Farrugia, 1999 ▶).

## Supplementary Material

Crystal structure: contains datablocks global, I. DOI: 10.1107/S1600536810010172/si2249sup1.cif

Structure factors: contains datablocks I. DOI: 10.1107/S1600536810010172/si2249Isup2.hkl

Additional supplementary materials:  crystallographic information; 3D view; checkCIF report

## Figures and Tables

**Table 1:** Selected bond lengths (Å)

N1—Ni1	2.1152 (13)
N2—Ni1	2.1095 (14)
O1—Ni1	2.1031 (10)

**Table 2:** Hydrogen-bond geometry (Å, °)

*D*—H⋯*A*	*D*—H	H⋯*A*	*D*⋯*A*	*D*—H⋯*A*
N1—H2⋯O1*W*	0.82 (3)	2.30 (3)	3.087 (2)	160 (2)
N2—H3⋯O2	0.82 (2)	2.43 (2)	3.0218 (19)	129.7 (19)
N1—H1⋯O2^i^	0.87 (3)	2.51 (3)	3.290 (2)	150 (2)
N2—H4⋯O2^ii^	0.83 (2)	2.26 (2)	3.092 (2)	177 (2)
O1*W*—H2*W*⋯O1^iii^	0.79 (2)	2.02 (2)	2.7999 (18)	173 (2)
O1*W*—H1*W*⋯O2^iv^	0.78 (2)	2.10 (2)	2.848 (2)	163 (3)

Symmetry codes: (i) 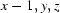; (ii) 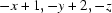; (iii) 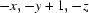; (iv) 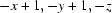.

**Fig. 1.**
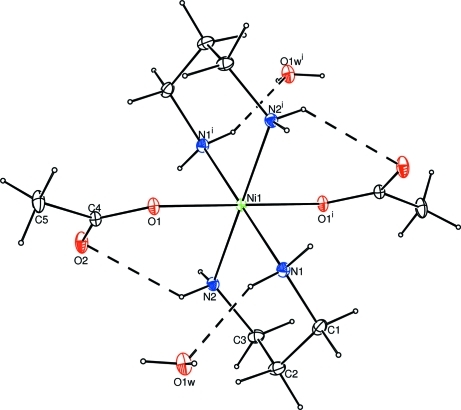
Molecular structure of (I), showing the atom-numbering scheme. Displacement ellipsoids are drawn at the 30% probability level. The intra- and intermolecular hydrogen bonds are shown as dashed lines. [Symmetry code: (i) -x, 2-y, -z.]

**Fig. 2.**
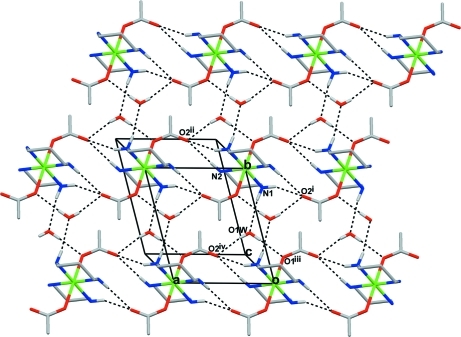
Perspective view of part of the crystal structure of (I), showing the formation of a hydrogen-bonded sheet built from R21(6), R22(12), R32(8) and R55(16) rings.

## supplementary crystallographic information

### Comment

The 1,3-Diaminopropane (tn) ligand behaves as a strong chelator in its metal complexes due to the formation of a stable six-membered ring. At the same time, it is a good H-bond donor due to the existence of amino groups (Sundberg *et al.*, 2001). Herein, we report the synthesis and structure of the title compound.

The molecular structure and atom-numbering scheme are shown in Fig. 1. The compound crystallizes in the space group P-1 with Z'=1/2. The nickel(II) ion is located on a symmetry center, and is coordinated by two O atoms from two identical carboxylate groups and four N atoms from two 1,3-diaminopropane ligands. The geometry around the nickel(II) ion (Table 1) is that of a slightly distorted octahedron, of which the equatorial plane (N1/N2/N1^i^/N2^i^) is formed by four amino N atoms [symmetry code:(i) -x, 2-y, -z]. The axial positions in the octahedron are occupied by two carboxylate O atoms (O1 and O1^i^). The 1,3-diaminopropane ligand shows chelating coordination behavior and displays a chair conformation [the puckering parameters (Cremer & Pople, 1975) are q_2_ = 0.0467 (17)Å , q_3_ = -0.5913 (18) Å, Q_T_ = 0.5930 (19) Å, φ = 349 (2)° and θ = 175.66 (16)°] in the equatorial direction.

The amino atom N1 in the molecule at (x, y, z) acts as a hydrogen-bond donor (Table 2) to atom O2^i^ so forming a C(6) (Bernstein *et al.*, 1995) chain running parallel to the [-100] direction. Amino atom N2 in the molecule at (x, y, z) acts as a hydrogen-bond donor to atom O2^ii^ so forming a C(6) chain running parallel to the [100] direction. The combination of N—H···O and O—H···O hydrogen bonds generates R_2_^1^(6), R_2_^2^(12), R_3_^2^(8) and R_5_^5^(16) rings parallel to the ab plane (Fig. 2).

### Experimental

Nickel(II) acetate (0.249 g, 1.0 mmol) was dissolved in methanol (25 ml). 1,3-diaminopropane(0.148 g, 2.0 mmol) were added and the mixture refluxed for 4 hours. The blue solution formed, which was filtered off, kept the filtrate for few days. Blue blocks were obtained from methanol.

### Refinement

All H atoms bound to C atoms were refined using a riding model, with C—H = 0.97Å and U_iso_(H) = 1.2U_eq_(C) for methylene C atoms and C—H = 0.96Å and U_iso_(H) = 1.5U_eq_(C) for methyl C atom. Water H atoms were located in difference maps and refined subject to a DFIX restraint of O—H = 0.83 (2) Å. Amino H atoms were located in difference maps and refined freely.

### Crystal data

**Table:**

[Ni(C_2_H_3_O_2_)_2_(C_3_H_10_N_2_)_2_]·2H_2_O	*Z* = 1
*M**_r_* = 361.09	*F*(000) = 194
Triclinic, *P*1	*D*_x_ = 1.404 Mg m^−^^3^
Hall symbol: -P 1	Mo *K*α radiation, λ = 0.71073 Å
*a* = 6.6268 (3) Å	Cell parameters from 5325 reflections
*b* = 7.8164 (3) Å	θ = 2.8–28.3°
*c* = 8.9123 (4) Å	µ = 1.17 mm^−^^1^
α = 73.933 (2)°	*T* = 296 K
β = 80.797 (3)°	Blocks, blue
γ = 75.323 (2)°	0.32 × 0.18 × 0.13 mm
*V* = 427.12 (3) Å^3^	

### Data collection

**Table:**

Bruker Kappa APEXII CCD area-detector diffractometer	2078 independent reflections
Radiation source: fine-focus sealed tube	2021 reflections with *I* > 2σ(*I*)
graphite	*R*_int_ = 0.025
phi and ω scans	θ_max_ = 28.3°, θ_min_ = 2.4°
Absorption correction: multi-scan (*SADABS*; Bruker, 2009)	*h* = −6→8
*T*_min_ = 0.775, *T*_max_ = 0.857	*k* = −10→10
7230 measured reflections	*l* = −11→11

### Refinement

**Table:**

Refinement on *F*^2^	Primary atom site location: structure-invariant direct methods
Least-squares matrix: full	Secondary atom site location: difference Fourier map
*R*[*F*^2^ > 2σ(*F*^2^)] = 0.024	Hydrogen site location: inferred from neighbouring sites
*wR*(*F*^2^) = 0.094	H atoms treated by a mixture of independent and constrained refinement
*S* = 1.02	*w* = 1/[σ^2^(*F*_o_^2^) + (0.0798*P*)^2^] where *P* = (*F*_o_^2^ + 2*F*_c_^2^)/3
2078 reflections	(Δ/σ)_max_ < 0.001
122 parameters	Δρ_max_ = 0.53 e Å^−^^3^
3 restraints	Δρ_min_ = −0.58 e Å^−^^3^

### Special details

**Table:**

Geometry. All esds (except the esd in the dihedral angle between two l.s. planes) are estimated using the full covariance matrix. The cell esds are taken into account individually in the estimation of esds in distances, angles and torsion angles; correlations between esds in cell parameters are only used when they are defined by crystal symmetry. An approximate (isotropic) treatment of cell esds is used for estimating esds involving l.s. planes.
Refinement. Refinement of *F*^2^ against ALL reflections. The weighted *R*-factor wR and goodness of fit *S* are based on *F*^2^, conventional *R*-factors *R* are based on *F*, with *F* set to zero for negative *F*^2^. The threshold expression of *F*^2^ > σ(*F*^2^) is used only for calculating *R*-factors(gt) etc. and is not relevant to the choice of reflections for refinement. *R*-factors based on *F*^2^ are statistically about twice as large as those based on *F*, and *R*- factors based on ALL data will be even larger.

### Fractional atomic coordinates and isotropic or equivalent isotropic displacement parameters (Å^2^)

**Table:**

	*x*	*y*	*z*	*U*_iso_*/*U*_eq_	
C1	−0.1844 (3)	0.7809 (3)	0.3153 (2)	0.0399 (4)	
H1A	−0.2579	0.6840	0.3658	0.048*	
H1B	−0.2714	0.8939	0.3353	0.048*	
C2	0.0196 (3)	0.7404 (3)	0.3857 (2)	0.0435 (4)	
H2A	0.1101	0.6333	0.3572	0.052*	
H2B	−0.0085	0.7106	0.4991	0.052*	
C3	0.1366 (3)	0.8935 (3)	0.3368 (2)	0.0407 (4)	
H3A	0.0437	1.0038	0.3579	0.049*	
H3B	0.2537	0.8620	0.3995	0.049*	
C4	0.3734 (2)	0.7432 (2)	−0.14011 (19)	0.0288 (3)	
C5	0.4393 (3)	0.6191 (3)	−0.2513 (3)	0.0509 (5)	
H5A	0.5557	0.6526	−0.3227	0.076*	
H5B	0.3242	0.6311	−0.3094	0.076*	
H5C	0.4798	0.4948	−0.1924	0.076*	
N1	−0.1533 (2)	0.7972 (2)	0.14486 (17)	0.0314 (3)	
H1	−0.274 (4)	0.816 (3)	0.110 (3)	0.047 (6)*	
H2	−0.073 (4)	0.700 (4)	0.135 (3)	0.047 (6)*	
N2	0.2158 (2)	0.9299 (2)	0.16906 (17)	0.0309 (3)	
H3	0.293 (3)	0.835 (3)	0.152 (2)	0.036 (5)*	
H4	0.293 (3)	1.004 (3)	0.145 (3)	0.038 (6)*	
O1	0.17922 (17)	0.79863 (15)	−0.11179 (14)	0.0319 (3)	
O2	0.5114 (2)	0.7828 (2)	−0.08414 (19)	0.0492 (4)	
O1W	0.0788 (2)	0.3937 (2)	0.1862 (2)	0.0462 (3)	
H1W	0.189 (3)	0.364 (4)	0.144 (3)	0.067 (8)*	
H2W	0.001 (3)	0.339 (3)	0.173 (3)	0.052 (7)*	
Ni1	0.0000	1.0000	0.0000	0.02287 (12)	

### Atomic displacement parameters (Å^2^)

**Table:**

	*U*^11^	*U*^22^	*U*^33^	*U*^12^	*U*^13^	*U*^23^
C1	0.0449 (9)	0.0370 (8)	0.0357 (9)	−0.0139 (7)	0.0100 (7)	−0.0089 (7)
C2	0.0616 (12)	0.0382 (9)	0.0291 (8)	−0.0117 (8)	−0.0034 (8)	−0.0055 (7)
C3	0.0528 (10)	0.0434 (9)	0.0297 (8)	−0.0125 (8)	−0.0085 (7)	−0.0110 (7)
C4	0.0239 (7)	0.0277 (7)	0.0356 (8)	−0.0050 (5)	0.0005 (6)	−0.0117 (6)
C5	0.0316 (9)	0.0667 (13)	0.0661 (13)	−0.0010 (8)	−0.0004 (8)	−0.0463 (11)
N1	0.0296 (7)	0.0305 (7)	0.0354 (7)	−0.0107 (6)	0.0009 (5)	−0.0087 (5)
N2	0.0276 (6)	0.0339 (7)	0.0330 (7)	−0.0053 (6)	−0.0044 (5)	−0.0118 (6)
O1	0.0227 (5)	0.0329 (6)	0.0449 (7)	−0.0050 (4)	0.0010 (4)	−0.0206 (5)
O2	0.0255 (6)	0.0670 (9)	0.0704 (10)	−0.0107 (6)	0.0005 (5)	−0.0441 (8)
O1W	0.0329 (7)	0.0489 (8)	0.0668 (9)	−0.0088 (6)	−0.0035 (6)	−0.0316 (7)
Ni1	0.01974 (16)	0.02373 (17)	0.02636 (18)	−0.00491 (10)	0.00030 (10)	−0.00948 (11)

### Geometric parameters (Å, °)

**Table:**

C1—N1	1.473 (2)	C5—H5B	0.9600
C1—C2	1.506 (3)	C5—H5C	0.9600
C1—H1A	0.9700	N1—Ni1	2.1152 (13)
C1—H1B	0.9700	N1—H1	0.87 (3)
C2—C3	1.515 (3)	N1—H2	0.82 (3)
C2—H2A	0.9700	N2—Ni1	2.1095 (14)
C2—H2B	0.9700	N2—H3	0.82 (2)
C3—N2	1.477 (2)	N2—H4	0.83 (2)
C3—H3A	0.9700	O1—Ni1	2.1031 (10)
C3—H3B	0.9700	O1W—H1W	0.777 (16)
C4—O2	1.2473 (19)	O1W—H2W	0.789 (15)
C4—O1	1.2572 (17)	Ni1—O1^i^	2.1031 (10)
C4—C5	1.515 (2)	Ni1—N2^i^	2.1095 (14)
C5—H5A	0.9600	Ni1—N1^i^	2.1152 (13)
			
N1—C1—C2	112.28 (14)	C1—N1—H1	109.3 (15)
N1—C1—H1A	109.1	Ni1—N1—H1	106.9 (16)
C2—C1—H1A	109.1	C1—N1—H2	104.5 (16)
N1—C1—H1B	109.1	Ni1—N1—H2	103.9 (16)
C2—C1—H1B	109.1	H1—N1—H2	114 (2)
H1A—C1—H1B	107.9	C3—N2—Ni1	118.74 (11)
C1—C2—C3	115.24 (16)	C3—N2—H3	108.3 (15)
C1—C2—H2A	108.5	Ni1—N2—H3	102.3 (15)
C3—C2—H2A	108.5	C3—N2—H4	112.4 (15)
C1—C2—H2B	108.5	Ni1—N2—H4	109.5 (16)
C3—C2—H2B	108.5	H3—N2—H4	104 (2)
H2A—C2—H2B	107.5	C4—O1—Ni1	132.72 (10)
N2—C3—C2	112.68 (15)	H1W—O1W—H2W	110 (2)
N2—C3—H3A	109.1	O1^i^—Ni1—O1	180.0
C2—C3—H3A	109.1	O1^i^—Ni1—N2^i^	91.58 (5)
N2—C3—H3B	109.1	O1—Ni1—N2^i^	88.42 (5)
C2—C3—H3B	109.1	O1^i^—Ni1—N2	88.42 (5)
H3A—C3—H3B	107.8	O1—Ni1—N2	91.58 (5)
O2—C4—O1	125.10 (14)	N2^i^—Ni1—N2	180.0
O2—C4—C5	118.96 (15)	O1^i^—Ni1—N1^i^	87.27 (5)
O1—C4—C5	115.95 (14)	O1—Ni1—N1^i^	92.73 (5)
C4—C5—H5A	109.5	N2^i^—Ni1—N1^i^	88.43 (6)
C4—C5—H5B	109.5	N2—Ni1—N1^i^	91.57 (6)
H5A—C5—H5B	109.5	O1^i^—Ni1—N1	92.73 (5)
C4—C5—H5C	109.5	O1—Ni1—N1	87.27 (5)
H5A—C5—H5C	109.5	N2^i^—Ni1—N1	91.57 (6)
H5B—C5—H5C	109.5	N2—Ni1—N1	88.43 (6)
C1—N1—Ni1	118.49 (10)	N1^i^—Ni1—N1	180.0
			
N1—C1—C2—C3	67.9 (2)	C4—O1—Ni1—N1	−127.02 (16)
C1—C2—C3—N2	−67.2 (2)	C3—N2—Ni1—O1^i^	51.20 (13)
C2—C1—N1—Ni1	−59.62 (18)	C3—N2—Ni1—O1	−128.80 (13)
C2—C3—N2—Ni1	58.12 (19)	C3—N2—Ni1—N1^i^	138.42 (13)
O2—C4—O1—Ni1	11.3 (3)	C3—N2—Ni1—N1	−41.58 (13)
C5—C4—O1—Ni1	−168.72 (14)	C1—N1—Ni1—O1^i^	−46.03 (14)
C4—O1—Ni1—N2^i^	141.34 (15)	C1—N1—Ni1—O1	133.97 (14)
C4—O1—Ni1—N2	−38.66 (15)	C1—N1—Ni1—N2^i^	−137.69 (14)
C4—O1—Ni1—N1^i^	52.98 (16)	C1—N1—Ni1—N2	42.31 (14)

Symmetry codes: (i) −*x*, −*y*+2, −*z*.

### Hydrogen-bond geometry (Å, °)

**Table:**

*D*—H···*A*	*D*—H	H···*A*	*D*···*A*	*D*—H···*A*
N1—H2···O1W	0.82 (3)	2.30 (3)	3.087 (2)	160 (2)
N2—H3···O2	0.82 (2)	2.43 (2)	3.0218 (19)	129.7 (19)
N1—H1···O2^ii^	0.87 (3)	2.51 (3)	3.290 (2)	150 (2)
N2—H4···O2^iii^	0.83 (2)	2.26 (2)	3.092 (2)	177 (2)
O1W—H2W···O1^iv^	0.79 (2)	2.02 (2)	2.7999 (18)	173 (2)
O1W—H1W···O2^v^	0.78 (2)	2.10 (2)	2.848 (2)	163 (3)

Symmetry codes: (ii) *x*−1, *y*, *z*; (iii) −*x*+1, −*y*+2, −*z*; (iv) −*x*, −*y*+1, −*z*; (v) −*x*+1, −*y*+1, −*z*.

## References

[bb1] Bernstein, J., Davis, R. E., Shimoni, L. & Chang, N.-L. (1995). *Angew. Chem. Int. Ed. Engl.* **34**, 1555–1573.

[bb2] Bruker (2009). *APEX2*, *SAINT* and *SADABS* Bruker AXS Inc., Madison, Wisconsin, USA.

[bb3] Cremer, D. & Pople, J. A. (1975). *J. Am. Chem. Soc.* **97**, 1354–1358.

[bb4] Farrugia, L. J. (1997). *J. Appl. Cryst.* **30**, 565.

[bb5] Farrugia, L. J. (1999). *J. Appl. Cryst.* **32**, 837–838.

[bb6] Sheldrick, G. M. (2008). *Acta Cryst.* A**64**, 112–122. 10.1107/S0108767307043930 18156677

[bb7] Sundberg, M. R., Kivekäs, R., Huovilainen, R. & Uggla, R. (2001). *Inorg. Chim. Acta*, **324**, 212–217.

